# Helmet interface increases lung volumes at equivalent ventilator pressures compared to the face mask interface during non-invasive ventilation

**DOI:** 10.1186/s13054-020-03216-7

**Published:** 2020-08-15

**Authors:** Kate C. Tatham, Matthew Ko, Lisa Palozzi, Stephen E. Lapinsky, Laurent J. Brochard, Ewan C. Goligher

**Affiliations:** 1 Section of Anaesthetics, Pain Medicine and Intensive Care, Department of Surgery and Cancer, Imperial College, 503 5th Floor Medical School Building, St Marys Hospital, Norfolk Place, London, W2 1PG UK; 2 Interdepartmental Division of Critical Care Medicine, University of Toronto, Toronto, Canada; 3 Intensive Care Unit, Mount Sinai Hospital, Toronto, Canada; 4 Critical Care Department, St Michael’s Hospital, Toronto, Canada; 5 Critical Care Department, Toronto General Hospital, Toronto, Canada

**Keywords:** Non-invasive ventilation, Helmet, Face mask, Acute hypoxemic respiratory failure

## Main text

Non-invasive ventilation (NIV) delivered by a helmet interface in acute respiratory distress syndrome (ARDS) has been associated with a lower rate of intubation, and mortality, compared to face mask NIV [1]. The mechanism accounting for this apparent benefit is uncertain; postulated mechanisms include more effective delivery of airway pressure due to better sealing of the interface and/or higher inspiratory flows.

**Fig. 1:**
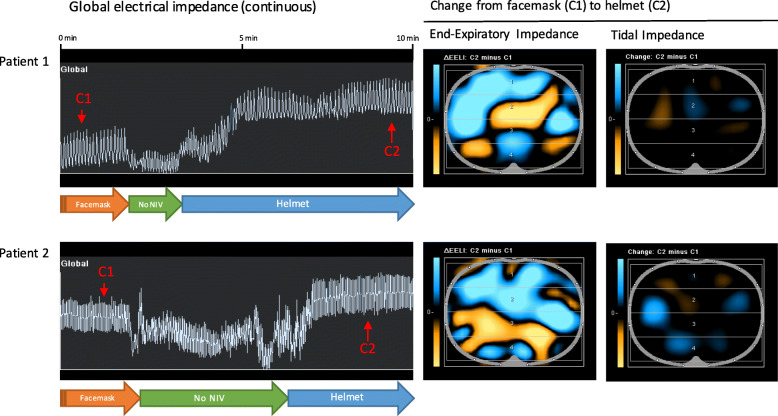
Changes in lung volume and the distribution of ventilation following transition from face mask to helmet interface for non-invasive ventilation. NB: orange = loss of ventilation; blue = gain of ventilation

During routine care of two patients with acute hypoxemic respiratory failure, we used electrical impedance tomography (‘EIT’, Draeger Pulmovista 500) to monitor ventilation while transitioning from face mask to helmet NIV. The transition to helmet NIV was a clinical decision prompted by worsening respiratory failure on face mask NIV, with the goal of avoiding intubation. EIT is a non-invasive imaging technique that permits visualization of lung volumes and the distribution of ventilation. Its high temporal resolution can detect rapid changes in lung volume during tidal ventilation and during adjustments to ventilator settings [2]. After calibration, EIT signals were recorded while the patients were ventilated on face mask NIV (Draeger V500 or BiPAP-Vision). We then exchanged the mask interface for a helmet interface (StarMed CaStar-R, Intersurgical), resuming ventilation at the same inspiratory and expiratory positive airway pressure (IPAP, EPAP) settings. During the transition, patients breathed without support at functional residual capacity. Global and regional end-expiratory lung impedance (EELI) and tidal impedance variation (TIV) were recorded throughout (Fig. 1). Twenty breaths were recorded under each condition (Table 1). Changes in end-expiratory lung volume were computed from changes in end-expiratory lung impedance by normalizing changes in lung impedance during tidal breathing to tidal volume measured by the ventilator [3]. Consent was obtained from the patients/legal representatives.

**Table 1:** Lung volumes and distribution of ventilation under face mask and helmet NIV

	Patient 1			Patient 2		
***Clinical history:***	*61-year-old female with metastatic small cell lung cancer admitted to the ICU for sepsis and acute hypoxemic respiratory failure*			*69-year-old female with acute myelogenous leukaemia admitted to ICU for acute hypoxemic respiratory failure*		
	**Face mask NIV**	**Transition (no NIV)**	**Helmet NIV**	**Face mask NIV**	**Transition (no NIV)**	**Helmet NIV**
Ventilator	Draeger V500	n/a	Draeger V500	BiPap Vision	n/a	Draeger V500
NIV setting (IPAP/EPAP), cm H_2_O	12/8	n/a	12/8	10/8	n/a	10/8
Tidal impedance variation (mean, SD)	1404 (93)	773 (202)	1163 (259)	2123 (259)	1724 (487)	2564 (245)
Tidal volume, ml (mean, SD)	392 (26)	216 (56)	325 (72)	375 (46)	310 (86)	462 (43)
End-expiratory lung impedance (mean, SD)	550 (126)	234 (83)	3022 (147)	3623 (128)	1956 (559)	5203 (212)
Computed end-expiratory lung volume above FRC, ml (mean, SE)	88 (34)	*Reference*	778 (38)	253 (134)	*Reference*	574 (140)
Proportion of tidal impedance in variation in dorsal lung region (%)	56	50	57	45	42	57
Respiratory rate (min^−1^)	24	31	25	33	32	34
Peripheral oxygen saturation (%)	92	n/a	96	93	n/a	95
Set FiO_2_*	0.55	n/a	0.4	0.5	n/a	0.4

*SD* standard deviation, *SE* standard error of the mean

*The FiO_2_ stated represents the requirement as determined by the respiratory therapists (RTs). FiO_2_ was titrated, depending on response, within approximately 1 h of the change in interface

Transition from face mask to helmet NIV was associated with a significant increase in EELI, predominantly in the ventral lung regions (Fig. 1). These changes in EELI were consistent with increases in end-expiratory lung volume (EELV) of 690 ml and 320 ml above FRC in the first and second patients, respectively (Table 1). Tidal impedance variation was redistributed dorsally in the second patient, possibly reflecting recruitment of previously non-ventilated lung regions. In both patients, oxygen saturations improved and FiO_2_ requirements decrease, on helmet NIV compared to face mask NIV (Table 1). The first patient required intubation after several hours on NIV via helmet; after 7 days of invasive mechanical ventilation, the patient recovered and was discharged to the ward. The second patient recovered after 24 h on helmet NIV and was discharged to the ward.

In summary, we observed that helmet NIV interface was associated with higher EELV compared to face mask NIV, even though the applied pressures were unchanged. This effect—and the potential lung recruitment that may accrue in some patients in consequence—might explain the apparent benefit of helmet NIV observed in a recent trial [1]. The mechanism accounting for this increase is unclear, potentially due to either a reduction in leak or a reduction in expiratory muscle activation. Studies are required to confirm this clinical finding and to delineate the responsible mechanisms. Of note, this report is not intended to suggest that helmet NIV should be applied with identical settings to face mask NIV, as previous investigators have shown that increases in pressure are required to unload the respiratory muscles because of lags in the pressurization of the helmet [4]. Rather, these results suggest the possibility that for any given pressure applied, helmet NIV may more effectively maintain EELV in comparison to the face mask interface.

## Data Availability

The datasets used and/or analysed during the current study are available from the corresponding author on reasonable request.

## Abbreviations

ARDS: Acute respiratory distress syndrome
EELI: End-expiratory lung impedance
EELV: End-expiratory lung volume
EIT: Electrical impedance tomography
EPAP: Expiratory positive airway pressure
IPAP: Inspiratory positive airway pressure
NIV: Non-invasive ventilation
TIV: Tidal impedance variation
